# 3 dimensional analysis of Hemimetameric Segmental Shift

**DOI:** 10.1186/1748-7161-10-S1-P20

**Published:** 2015-01-19

**Authors:** Toshiki Saito, Noriaki Kawakami, Taichi Tsuji, Tetsuya Ohara, Yoshitaka Suzuki, Ayato Nohara, Ryoji Tauchi, Kousuke Takimura

**Affiliations:** 1 Dept. Orthop & Spine Surg, Meijo Hospital, Japan

## Introduction

Hemimetameric Segmental Shift (HMMS) is defined as a hemivertebral deformation in which two or more hemivertebra exists on both left and right sides of the spine, where the hemivertebras are separated by at least 1 normal vertebra. 3D-CT analysis done by Kawakami et al on congenital scoliosis patients proved the existence of mismatch among the anterior and posterior segments, where they coined this phenomenon as discordant anomaly. The purpose of this study is to analyze the morphology and determine clinical features of HMMS 3 dimensionally.

## Methods

HMMS existed in 32 (6.6%) out of 483 patients diagnosed of congenital scoliosis at the respective institution between the years 1998-2013. Of the 32 there were 16 males and 16 females. Average age at the time of their first visit was 6 years 3 months. 3D-CT imaging was done to 30 patients older than 2 years old, with an average age of 9 years 8 months. Using 3D-CT imaging, these 30 patients were classified with posterior elements.

## Results

Number of patients for each number of hemivertebra present was 21 patients for 2 hemivertebra, 7 patients for 3 hemivertebra, and 2 patients for 4 hemivertebra. Patients with 2 hemivertebra were most common to have hemivertebra in the thoracolumbar spine, while patients with 3 or more hemivertebra was most common to have hemivertebra in the thoracic spine.

Analysis using 3D-CT images classified patients into two categories, where malformation exists at an equal level in anterior and posterior sides (unison HMMS) and malformation existing at nonequal levels (discordant HMMS). 9 patients were classified as unison HMMS where all 9 of these patients had 2 hermivertebra. Average number of malformed vertebra in this group was 4.6. On the other hand, 21patients were classified as having discordant HMMS, where 12 patients had 2 hemivertebra, 7 had 3 hemivertebra and 2 had 4 hemivertebra. Average numbers of malformed vertebras were 6.9 in this group, with greater number of malformed vertebras than in the unison HMMS group.

## Conclusion

HMMS was categorized into unison and discordant classifications. Discordant HMMS existed among 21 patients out of 30 (70%), where all patients with more than 3 hemivertebra were of this type. Out of the 21 patients with 2 hemivertebra, 12 patients (57%) had discordant HMMS. Operation on HMMS patients should be done with extreme care with careful analysis of bone model and radiological images in order to correctly decide on their spinal levels.

